# Effect of Dark Triad on gratitude: the roles of belief in a just world and life history strategy

**DOI:** 10.3389/fpsyg.2026.1925957

**Published:** 2026-08-28

**Authors:** Yaoguo Geng, Peiyan Zeng, Liping Shi, Wenjing Jin

**Affiliations:** 1 School of Marxism, Zhengzhou University, Zhengzhou, China; 2 School of Kinesiology and Physical Education, Zhengzhou University, Zhengzhou, China; 3 School of Journalism and Communication, Henan University of Technology, Zhengzhou, China; 4 School of Marxism, Henan University of Technology, Zhengzhou, China

**Keywords:** adolescent, belief in a just world, Dark Triad, gratitude, life history strategy

## Abstract

**Introduction:**

Grounded in belief in a just world theory and life history theory, this study examined the associations between Dark Triad traits and gratitude among Chinese adolescents.

**Methods:**

A total of 734 middle school students (M = 13.71, SD = 1.03; 361 males, 373 females) were recruited via a cross-sectional design, and all participants completed the Short Dark Triad (SD3), Mini-K Scale, Adolescent Gratitude Scale (AGS), and Belief in a Just World Scale (BJWS).

**Results:**

Correlation analyses indicated that Machiavellianism, psychopathy, and narcissism were all negatively correlated with gratitude. Regression-based mediation analyses further showed that: belief in a just world mediated the associations of Machiavellianism and psychopathy with gratitude, respectively; life history strategy only mediated the association between psychopathy and gratitude; furthermore, belief in a just world and life-history strategy chain-mediated the associations of Machiavellianism and psychopathy with gratitude.

**Conclusion:**

These findings delineate the differing patterns of gratitude associated with distinct Dark Triad traits and illuminate the indirect pathways underlying these associations.

## 1 Introduction

Gratitude is a moral emotion and core prosocial virtue that strengthens interpersonal trust, emotional bonding, and reciprocal cooperation across the lifespan (Algoe, 2012; McCullough et al., 2002). For adolescents, gratitude carries unique developmental significance, as it critically shapes moral reasoning, peer adaptation, and long-term socioemotional adjustment (Tudge and de Lucca Freitas, 2017; Wong et al., 2024). Early adolescence, the age range of the present sample, represents a critical formative window for gratitude. Youth gradually develop sophisticated social-cognitive capacities to interpret others’ benevolent intentions, and stable trait gratitude crystallizes during this phase (Hussong et al., 2019; Li et al., 2025). A 2025 longitudinal study of Chinese early adolescents further confirmed that dispositional gratitude prospectively predicts better mental wellbeing and fewer externalizing behavioral problems over two school years (Li et al., 2025). Therefore, unpacking personality antecedents of gratitude and their underlying psychological pathways among early adolescents holds important theoretical and practical implications for youth moral education.

Some evidence has been accumulated that gratitude and personality traits are related. For example, among the Big Five personality factors, agreeableness shows the strongest positive association with gratitude, while neuroticism is negatively associated to gratitude (Wood et al., 2008). From a bandwidth-fidelity trade-off perspective, narrow dark personality constructs such as the Dark Triad provide greater predictive specificity for social outcomes than broad Big Five dimensions (Greitemeyer, 2022). The Dark Triad refers to three interrelated but empirically distinct antisocial personality traits: Machiavellianism, narcissism, and psychopathy (Paulhus and Williams, 2002). Despite their unique features, the three traits share a common core of dishonesty, callousness, interpersonal manipulation, and low empathy (Furnham et al., 2013). Specifically, Machiavellianism is defined by strategic self-interest and cynical manipulation of others to advance personal goals (Malesza and Ostaszewski, 2016); narcissism is characterized by grandiose self-views, a pervasive sense of entitlement, and a need for admiration (Jones and Paulhus, 2014); and psychopathy entails affective callousness, impulse, and disregard for social norms (Geng et al., 2018). Cross-cultural studies consistently show that individuals with high Dark Triad traits prioritize short-term self-interest and exploit others’ kindness for instrumental gains, impairing their prosocial and emotional functioning (Jonason and Schmitt, 2012; Puthillam et al., 2021). Adolescent-based research further validates the negative impact of Dark Triad traits on prosocial behavior. Giancola et al. (2022) found that the Dark Triad negatively predicted late adolescents’ altruistic and equitable prosocial engagement, with trait emotional intelligence mediating this relationship. A recent longitudinal study of Chinese adolescents similarly revealed that elevated Dark Triad traits reduce prosocial behaviors, whose rare prosocial acts are motivated by self-interest rather than genuine benevolence (Xue et al., 2026). Beyond general prosocial deficits, the Dark Triad traits also inhibit pro-environmental behavior by lowering biospheric value orientation and reducing concern for collective ecological benefits (Giancola et al., 2023; Ucar et al., 2023). Most importantly, dark personality traits induce biased social cognition that hinders gratitude development. Individuals with high Dark Triad traits tend to distrust others’ benevolent intentions and perceive interpersonal interactions as manipulative, failing to adequately recognize and appreciate others’ prosocial efforts (Abell et al., 2016). This stable cognitive bias accounts for their lower gratitude levels in daily social interactions.

Despite these consistent negative associations between the Dark Triad traits and broad pro-sociality, targeted research linking the Dark Triad specifically to gratitude remains limited, especially for early adolescents. Puthillam et al. (2021) examined the associations between the Dark Triad and state/trait gratitude across adult samples, documenting significant negative correlations for Machiavellianism and psychopathy, while narcissism displayed non-significant links. This divergent pattern across subdimensions is attributed to narcissism’s “brighter” agentic component relative to the overtly exploitative and unempathic features of Machiavellianism and psychopathy (Rauthmann and Kolar, 2013). Follow-up work with Chinese middle school students replicated the negative Machiavellianism–gratitude pathway, demonstrating that Machiavellianism indirectly diminishes academic wellbeing via reduced gratitude (Yang et al., 2022). However, nearly all existing gratitude-focused Dark Triad research relies on adult, undergraduate, or late adolescent samples. Very few investigations center on early adolescence, a developmental stage during which dark personality dispositions and gratitude both develop and stabilize. Prior literature therefore lacks clear evidence on whether the Dark Triad–gratitude association replicates in this younger group and manifests unique developmental patterns among middle school students. Building on this cumulative cross-sectional and longitudinal evidence, we hypothesize that the Dark Triad traits, especially Machiavellianism and psychopathy, will be negatively associated with adolescent gratitude.

Belief in a just world (BJW) refers to a core social-cognitive schema through which individuals hold the conviction that life outcomes correspond to deserved effort and conduct (Lerner, 1965). Robust empirical data confirm positive concurrent and longitudinal associations between BJW and gratitude, interpersonal trust, altruism, and subjective wellbeing (Dzuka and Dalbert, 2006; Wang et al., 2021). Functionally, BJW fosters perceptions of the social environment as predictable and fair, enhancing perceived control and individuals’ willingness to reciprocate others’ kindness (Peng et al., 2019). In contrast, individuals scoring high on Dark Triad traits tend to adopt chaotic, unfair worldviews, rejecting reciprocal justice norms (Sai et al., 2020; Sherman et al., 2013). A 2026 longitudinal study of Chinese adolescents further verified bidirectional associations between Dark Triad traits and diminished personal/general BJW, showing that high antagonistic personalities erode the prosocial benefits of stable justice beliefs (Xue et al., 2026). Taken together, these findings suggest that BJW may serve as a plausible mediator linking Dark Triad traits to gratitude, yet no existing research has examined this indirect pathway specifically in early adolescent populations.

Life history theory frames individual resource allocation tradeoffs across growth, mating, and cooperative investment along a slow–fast strategic continuum (Kaplan and Gangestad, 2015; Nettle, 2010). Fast life history strategy prioritizes immediate, self-serving rewards, impulsivity, and shallow interpersonal bonds (Puthillam et al., 2021; Wang et al., 2017), whereas slow life history strategy prioritizes delayed gratification, long-term reciprocity, and sustained prosocial engagement (Fang, 2020; Sherman et al., 2013). A key line of life history research focuses on the link between life history strategy and dark personality traits. Previous studies have shown that Machiavellianism and psychopathy are consistently associated with a fast life history strategy, while narcissism shows only a weak association with fast strategic tendencies (Jonason et al., 2017). Puthillam et al. (2021) theoretically proposed life history strategy as a potential explanatory variable for the Dark Triad–gratitude relationship, yet their adult dataset lacked formal mediation testing; to date, no empirical study has verified this mediating pathway among early adolescents.

Crucially, the BJW is closely linked to the formation of life history strategy, suggesting a sequential cognitive-behavioral pathway that may operate as a chain mediator between the Dark Triad traits and gratitude. Multiple recent Chinese adolescent and college student studies demonstrate that BJW is a robust predictor of life history strategy: adolescents who perceive the world as fair and predictable tend to adopt slower, cooperative life strategies, whereas low BJW is associated with perceptions of environmental instability and a preference for fast, opportunistic resource tradeoffs (Lin et al., 2022; Meng et al., 2019). Callan et al. (2014) further experimentally validated that manipulative, untrustful worldviews reduce delay discounting and preference for long-term reciprocal exchange—core psychological features incompatible with gratitude. However, the evidence for these pathways derives almost exclusively from adult and late adolescent samples, leaving early adolescents unexamined—despite this being a critical period for the consolidation of both dark personality and gratitude. Moreover, prior research has tested only isolated correlations or single-mediator models, without integrating both BJW and life history strategy as sequential mediators; nor has it systematically compared how different Dark Triad subdimensions—Machiavellianism, psychopathy, and narcissism—may operate through these pathways. Building upon established pairwise associations documented in prior adult and late adolescent research, the present study tests a unified chain mediation model in a sample of Chinese early adolescents aged 12–15 years. Specifically, it is hypothesized that the Dark Triad traits influence gratitude through the BJW and the life history strategy.

Drawing on the theoretical frameworks reviewed above, the present study proposes four hypotheses:

Hypothesis 1 (H1): Machiavellianism, psychopathy and narcissism are all negatively associated with gratitude.

Hypothesis 2 (H2): BJW plays a significant mediating role in the negative relationships of Machiavellianism and psychopathy with gratitude. However, it does not play a significant mediating role for narcissism.

Hypothesis 3 (H3): Life history strategy plays a significant mediating role in the negative relationships of Machiavellianism and psychopathy with gratitude, whereas this mediating effect is not hypothesized for narcissism.

Hypothesis 4 (H4): The negative associations of Machiavellianism and psychopathy with gratitude are chain-mediated through BJW and life history strategy, whereas this chain mediation is not expected for narcissism.

## 2 Method

### 2.1 Sample and procedure

A convenience sampling approach was employed, with intact classes adopted as the cluster sampling unit. Participants were recruited from a total of 27 classes across three middle schools in Henan Province, China, and 800 students were initially enrolled. After excluding invalid questionnaires with substantial missing responses, the final valid sample consisted of 734 adolescents (effective response rate = 91.75%), aged 12–15 years (M = 13.71, SD = 1.03). Among them, there were 373 females (50.82%) and 361 males (49.18%); 30 only children (4.09%) and 704 non-only children (95.91%); 158 participants with urban household registration (21.53%) and 576 participants with rural household registration (78.47%). An *a priori* power analysis conducted via G*Power (power = 0.95, effect size = 0.15) indicated that the minimum required sample size was 111 for correlation analyses and 119 for multiple linear regression. The attained valid sample of 734 guaranteed adequate statistical power for all planned analyses.

The recruitment was conducted from October to November 2021. The research team obtained school consent first, then selected classes randomly. Teachers informed parents and students of the research purpose, voluntary participation principle, strict confidentiality and anonymity measures, potential risks, and corresponding compensation via WeChat groups to obtain parental consent. Under the guidance of psychology professors, standardized testing of participants was completed by uniformly trained master’s students majoring in psychology, who administered paper-based questionnaires after class breaks with unified guidance and on-site collection.

### 2.2 Measures

The Short Dark Triad (SD3; Geng et al., 2015; Jones and Paulhus, 2014) is a brief 27-item self-report scale used to measure the Dark Triad. Participants were asked how much they agreed (1 = strongly disagree; 5 = strongly agree) with statements such as, “It’s not wise to tell your secrets.” (i.e., Machiavellianism), “I like to get acquainted with important people.” (i.e., narcissism), and “People often say I’m out of control” (i.e., psychopathy). One sensitive item (“I enjoy having sex with people I hardly know”) was developmentally adapted to “I like to establish romantic relationships with strangers” for 12–15-year-old adolescent participants. In confirmatory factor analysis of the full scale, the measurement model demonstrated acceptable fit (*χ*^2^/df = 4.60, CFI = 0.91, TLI = 0.91, RMSEA = 0.07), and this adapted item exhibited a satisfactory standardized factor loading (>0.60) on its intended factor. Higher scores indicate higher levels of the corresponding Dark Triad traits. In the present study, the Cronbach’s *α* for Machiavellianism, narcissism, and psychopathy were 0.80, 0.71, and 0.75, respectively.

The Adolescent Gratitude Scale (AGS; He et al., 2012) is a brief 23-item self-report scale used to measure gratitude. Participants were asked how much they agreed (1 = strongly disagree; 5 = strongly agree) with statements such as, “I always repay my teachers’ instructions and care in some way.” Higher scores indicate higher levels of gratitude. The Cronbach’s *α* was 0.90.

Life history strategy was assessed using the Chinese version of the Mini-K Scale (Figueredo et al., 2006; Chen and Chang, 2016). This 20-item self-report scale measures individual differences in life history speed along the fast–slow continuum. Participants were asked to rate their agreement with each item on a 7-point Likert-type response scale (I often find the positive side of an event in its negative aspects.). Higher scores indicate a slower life history strategy. The Cronbach’s *α* was 0.87.

The Belief in a Just World Scale (BJWS; Dalbert, 1999; Su et al., 2012) consists of 13 items along a 6-point Likert-type response scale (1 = strongly disagree; 6 = strongly agree), assessing two factors: the personal belief in a just world (PBJW: I believe that most of the things that happen in my life are fair) and the general belief in a just world (GBJW: To a large extent, I believe that people get what they deserve). Higher scores indicate the stronger belief in a just world. The Cronbach’s *α* were 0.83 and 0.85, respectively.

### 2.3 Data analysis

All valid questionnaires were entered into SPSS software, and statistical analyses of the collected data were performed using SPSS 25.0. Specifically, Pearson correlation analysis was adopted to calculate the correlations among the main variables; independent samples *t*-tests were conducted to examine gender differences in the variables; and the chain mediation model was tested by employing the Process macro (Model 6) for SPSS.

## 3 Results

### 3.1 Correlations

The results are shown in Table 1. Machiavellianism, psychopathy, and narcissism (i.e., the Dark Triad traits) were all significantly negatively correlated with gratitude, supporting Hypothesis 1. Machiavellianism and psychopathy were significantly negatively correlated with BJW and life history strategy, while narcissism was negatively correlated only with BJW. In addition, BJW was positively associated with life-history strategy and gratitude, and life-history strategy was positively associated with gratitude.

**Table 1:** Zero-order correlations among study variables.

Variable	1 SD3	2 P	3 N	4 M	5 BJW	6 Mini-K
1. SD3	–					
2. P	0.82^**^	–				
3. N	0.70^**^	0.44^**^	–			
4. M	0.80^**^	0.44^**^	0.33^**^	–		
5. BJW	−0.22^**^	−0.30^**^	−0.04	−0.15^**^	–	
6. Mini-K	−0.21^**^	−0.31^**^	−0.05	−0.11^**^	0.48^**^	–
7. G	−0.38^**^	−0.50^**^	−0.21^**^	−0.16^**^	0.55^**^	0.51^**^

^**^p < 0.01; SD3, Dark Triad; P, Psychopathy; N, Narcissism; M, Machiavellianism; BJW, Belief in a Just World; Mini-K, Life History Strategy; G, Gratitude.

### 3.2 Gender differences

Gender differences and descriptive statistics for the variables are reported in Table 2. Significant gender differences were observed for Dark Triad traits (Machiavellianism, narcissism, psychopathy), life history strategy, and gratitude. Specifically, males scored significantly higher on Dark Triad traits than females, whereas females exhibited higher Mini-K and gratitude scores than males.

**Table 2:** Gender differences on the main variables.

Variable	Male	Female	*t*	Cohen’s *d*
	*n* (361)	*n* (373)		
SD3	74.56 ± 13.79	65.65 ± 13.34	8.92^**^	0.66
P	21.67 ± 6.17	18.28 ± 6.35	7.34^**^	0.54
N	25.28 ± 5.01	23.34 ± 4.43	5.56^**^	0.41
M	27.69 ± 6.90	24.02 ± 6.77	7.29^**^	0.54
BJW	48.85 ± 13.12	49.47 ± 11.08	−0.68	−0.05
Mini-K	95.07 ± 20.37	97.94 ± 16.14	−2.13^*^	−0.16
G	82.82 ± 15.03	87.29 ± 14.10	−4.13^**^	−0.31

^*^p < 0.05, ^**^p < 0.01; Gender: 1, male; 0, female.

### 3.3 Testing for the proposed model

**Figure 1:**
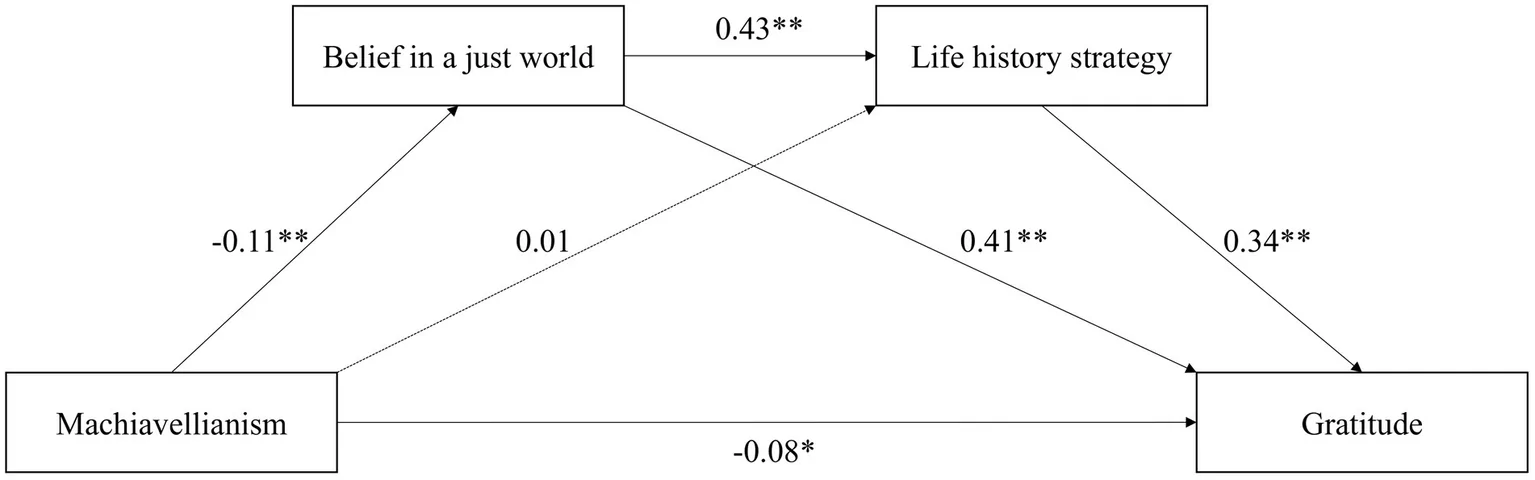
The model of the mediating effect (Machiavellianism).

Regression analysis showed that after controlling for gender and age, the total effect of Machiavellianism on gratitude was −0.14 (*p* < 0.01). Machiavellianism negatively predicted gratitude (*β* = −0.08, *p* < 0.05) and BJW (*β* = −0.11, *p* < 0.01). BJW positively predicted life history strategy (*β* = 0.43, *p* < 0.01) and gratitude (*β* = 0.41, *p* < 0.01). Life history strategy positively predicted gratitude (*β* = 0.34, *p* < 0.01). Detailed results are presented in Table 3 and Figure 1.

**Table 3:** The results of chain mediation analysis (Machiavellianism).

Model	Outcome	Predictor	*R*	*R* ^2^	*F*	*β*	*t*
Model 1	BJW	M	0.11	0.01	3.04^*^	−0.11	−2.99^**^
		Age				0.01	0.21
		Gender				0.06	0.76
Model 2	Mini-K	M	0.45	0.21	46.37^**^	0.01	0.30
		BJW				0.43	13.28^**^
		Age				0.07	2.00^*^
		Gender				−0.11	−1.68
Model 3	G	M	0.65	0.42	103.44^**^	−0.08	−2.54^*^
		BJW				0.41	12.49^**^
		Mini-K				0.34	10.28^**^
		Age				−0.04	−1.42
		Gender				−0.23	−3.94^**^

^*^p < 0.05, ^**^p < 0.01.

The results indicated two significant indirect effects. First, the indirect effect of Machiavellianism on gratitude through BJW was −0.05 (95%CI = [−0.09, −0.01]), accounting for 35.71% of the total effect. Second, the chain indirect effect of Machiavellianism on gratitude through BJW and life history strategy was −0.02 (95%CI = [−0.03, −0.01]), accounting for 14.29% of the total effect. Finally, the total indirect effect accounted for 50.00% of the total effect.

**Figure 2:**
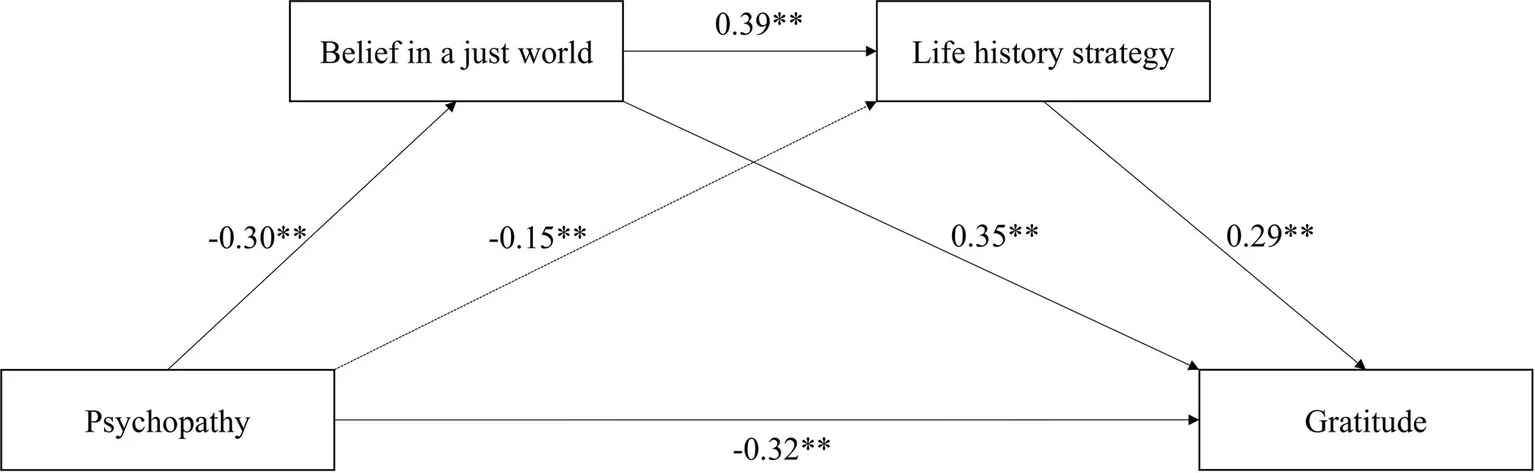
The model of the mediating effect (Psychopathy).

Regression analysis showed that after controlling for gender and age, the total effect of psychopathy on gratitude was −0.50 (*p* < 0.01). Psychopathy negatively predicted gratitude (*β* = −0.32, *p* < 0.01), BJW (*β* = −0.30, *p* < 0.01) and life history strategy (*β* = −0.15, *p* < 0.01). BJW positively predicted life history strategy (*β* = 0.39, *p* < 0.01) and gratitude (*β* = 0.35, *p* < 0.01). Life history strategy positively predicted gratitude (*β* = 0.29, *p* < 0.01). Detailed results are shown in Table 4 and Figure 2.

**Table 4:** The results of chain mediation analysis (Psychopathy).

Model	Outcome	Predictor	*R*	*R* ^2^	*F*	*β*	*t*
Model 1	BJW	P	0.28	0.08	21.21^**^	−0.30	−7.97^**^
		Age				0.02	0.42
		Gender				0.15	2.04^*^
Model 2	Mini-K	P	0.46	0.23	52.59^**^	−0.15	−4.46^**^
		BJW				0.39	11.69^**^
		Age				0.07	2.05^*^
		Gender				−0.03	−0.45
Model 3	G	P	0.71	0.50	143.34^**^	−0.32	−11.11^**^
		BJW				0.35	11.43^**^
		Mini-K				0.29	9.08^**^
		Age				−0.03	−1.15
		Gender				−0.11	−2.04^*^

^*^p < 0.05, ^**^p < 0.01.

The results indicated three significant indirect effects. First, indirect effect of psychopathy on gratitude through BJW was −0.10 (95%CI = [−0.14, −0.07]), accounting for 20.00% of the total effect. Second, the indirect effect of psychopathy on gratitude through life history strategy was −0.04 (95%CI = [−0.07, −0.02]), accounting for 10.00% of the total effect. Third, the chain indirect effect of psychopathy on gratitude through BJW and life history strategy was −0.04 (95%CI = [−0.05, −0.02]), accounting for 10.00% of the total effect. Overall, the total indirect effect accounted for 36.00% of the total effect.

The standardized indirect effects between narcissism and gratitude were non-significant, all 95%CIs were including 0. The results of the mediation analysis indicated that Hypotheses 2, 3, and 4 were all partially supported.

## 4 Discussion

Based on belief in a just world theory and the life history theory, the present study tested a chain mediation model to explore the relationship between the Dark Triad traits and gratitude. This study results suggest that adolescents with high Dark Triad traits reported lower gratitude. BJW and life history strategy played chain mediating roles in the relationship between the Dark Triad traits (except narcissism) and gratitude.

The results showed that all dark traits were negatively associated with gratitude (H1). This core finding largely replicates the cross-sectional evidence from adult samples, in which Puthillam et al. (2021) confirmed that Machiavellianism and psychopathy robustly predicted reduced trait gratitude, whereas narcissism yielded non-significant correlations with gratitude among adults. Importantly, this theoretical pattern is extended to early adolescent cohorts in the present study, with nuanced developmental differences further considered. Given that maladaptive narcissistic tendencies (e.g., hypersensitivity, willfulness) have been found to decline across the lifespan (Chopik and Grimm, 2019), it follows that these features are likely relatively pronounced during adolescence. Maladaptive narcissistic features in adolescents are predominantly characterized by egocentric cognition, interpersonal dominance, and lack of prosocial attunement, which are associated with a reduced capacity to recognize and appreciate others’ kindness. This renders adolescence a developmental stage in which the negative association between narcissism and gratitude is particularly salient. In contrast, adult narcissism increasingly incorporates agentic adaptive components (e.g., autonomy; Chopik and Grimm, 2019), which may attenuate its negative emotional correlates, potentially coinciding with a non-significant or attenuated association with gratitude in adult populations.

In addition to narcissism, adolescents high in Machiavellianism reported less gratitude, as their interpersonal manipulation and utilitarian social cognition lead them to interpret others’ kindness merely as instrumental exchanges rather than genuine goodwill, thereby diminishing positive emotional arousal triggered by receiving benefits (Müller et al., 2021; Malesza and Ostaszewski, 2016). Psychopathy also predicted decreased gratitude, stemming from core deficits in affective empathy, emotional callousness, and impulsive antisocial tendencies (Geng et al., 2018). Individuals with prominent psychopathic traits adopt rigid, coercive communication strategies and lack interpersonal flexibility; they even employ aggressive humor to seek dominance without regard for others’ feelings, which fundamentally impairs their ability to perceive and reciprocate kindness from peers (Dionigi et al., 2022; Duradoni et al., 2023).

Results confirmed that BJW mediated the relationships between Machiavellianism/psychopathy and gratitude (H2). In line with prior research linking the Dark Triad traits to weakened just-world beliefs (Jonason et al., 2015; Song et al., 2018), individuals with high antagonistic personality traits hold low BJW, which further suppresses gratitude formation. Consistent with Lerner’s (1965) classic just-world hypothesis, individuals with strong BJW tend to believe that efforts and kindness will be fairly rewarded, motivating them to recognize external positive support and experience grateful emotions (Ellard et al., 2016; Lin et al., 2022; Lu, 2020). Conversely, adolescents scoring high on Machiavellianism and psychopathy perceive the social world as unfair and unpredictable. They take interpersonal favors for granted due to self-serving value orientations, lose the cognitive foundation for reciprocal gratitude, and rarely experience grateful emotions after receiving help (Jonason et al., 2015). Specifically, individuals high in psychopathy exhibit unstable lifestyles and antisocial tendencies, ignoring others’ wellbeing and demonstrating a high propensity for betrayal (Deutchman and Sullivan, 2018); their low BJW erodes the cognitive basis for recognizing others’ kindness, which explains why they fail to respond with goodwill even when receiving favors (Malesza, 2020). Meanwhile, those high in Machiavellianism frame social interactions as interest-based exchanges (Geng et al., 2014), and it is these traits that underpin their low BJW and negate the belief in reciprocal reward. This mediating pathway is highly consistent with the longitudinal research on Chinese adolescents by Xue et al. (2026), which verified that Dark Triad traits disrupt the coherence between personal and general BJW and further reduce adolescents’ prosocial engagement. The present study extends this cognitive explanatory framework to the specific affective outcome of gratitude, broadening the empirical application of just world theory in research on adolescent prosocial emotional development.

Life history strategy mediated the relationship between psychopathy and gratitude (H3). According to life history theory, personality traits reflect adaptive calibrations to environmental conditions. Therefore, the personality traits of individuals are consistent with their life history strategies, that is, the behavioral patterns are related to the life history strategies adopted (Figueredo et al., 2006). Individuals with high psychopathy tend to adopt a fast life history strategy in order to adapt to the environment (Sai et al., 2020). A fast life history strategy is associated with lower prosocial behavior, diminished positive emotional engagement, and a greater tendency to take others’ contributions for granted (Sherman et al., 2013; Fang, 2020). By contrast, no such mediating effect emerged for Machiavellianism. Jonason et al. (2017) suggested that the modest correlation between Machiavellianism and fast life history strategy is entirely attributable to its shared variance with psychopathy; Machiavellianism itself carries no independent predictive effect on life history strategy. This result validates the differentiated evolutionary underpinnings of the Dark Triad subdimensions in adolescent samples, thereby refining the oversimplified unitary Dark Triad-life history framework that characterizes most adult-oriented research.

This study also found that Machiavellianism and psychopathy were associated with gratitude through the chain mediation of BJW and life history strategy (H4). This chain mechanism expands existing separate mediation findings by revealing a continuous cognitive-evolutionary pathway among early adolescents. Individuals with high Machiavellianism and psychopathy tend to hold low BJW, perceiving the world as fundamentally unfair and unpredictable. As relevant studies have shown, BJW is an important cognitive antecedent for predicting individuals’ life history strategy (Meng et al., 2019; Lin et al., 2022). Low BJW breaks the internal psychological contract that long-term efforts will be rewarded (Lin et al., 2022). When individuals perceive the environment as chaotic and uncontrollable due to low BJW, their ability to delay gratification and their investment in future development are reduced, thus forming a fast life history strategy focused on immediate benefits and avoidance of deep interpersonal bonds (Hafer et al., 2005; Meng et al., 2019). A fast life history strategy conflicts inherently with the core attributes of gratitude (valuing reciprocity, appreciating others’ kindness, and maintaining enduring social ties), thereby lowering adolescents’ dispositional gratitude levels.

Nevertheless, the chain mediation path was non-significant for narcissism. Two complementary explanations account for this null result. First, narcissism centers on self-enhancement and admiration-seeking motives rather than cynical disbelief in worldly fairness, so it cannot trigger the BJW-life history sequential cognitive pathway (Jones and Paulhus, 2014). Second, measurement limitations of the SD3 scale contribute to this non-significant effect: the Short Dark Triad mainly captures grandiose narcissism and fails to fully assess vulnerable, maladaptive narcissistic facets, which weakens its predictive power on internal cognitive and emotional outcomes. This measurement constraint has been widely documented in psychometric reviews of the SD3 scale (Geng et al., 2015).

## 5 Conclusion

This study examined the relationship between the Dark Triad and gratitude and revealed that BJW and life history strategy serve as key chain mediators in this relationship. Specifically, BJW mediates the relationship between Machiavellianism/psychopathy and gratitude, with lower BJW associated with both higher personality trait levels and lower gratitude. By contrast, life history strategy mediated the relationship only between psychopathy and gratitude. Importantly, the present study identified a chain mediating pathway from BJW to life history strategy. This indicates that individuals with dark personality traits hold a weaker perception that the world is just and orderly, and further adopt a fast life history strategy oriented toward immediate gratification. Ultimately, these two factors (low BJW and fast life history strategy) are associated with lower gratitude through this dual cognitive-strategic process. These findings provide an integrated theoretical framework that combines cognitive and evolutionary perspectives for understanding how dark personality traits shape positive social emotions.

## 6 Limitations and future research directions

Despite its contributions, this study has several limitations. First, all data were collected via participants’ self-reports, which may introduce social desirability bias. Second, the cross-sectional study design cannot establish causal relationships or clarify the temporal sequence of variables; accordingly, the hypothesized chain mediation model only serves as a statistical decomposition of concurrent correlations. Third, using total scores for the Belief in a Just World Scale and the Mini-K may obscure distinct correlational patterns among their respective subdimensions. Fourth, one SD-3 item was developmentally reworded for adolescent participants. Although this revised item demonstrated satisfactory factor loading in the present sample, findings derived from it should be interpreted with caution, and cross-sample validation is warranted. Future research could address these limitations by incorporating objective measures (e.g., behavioral experiments and physiological measurements), adopting longitudinal designs, employing SEM at the subdimension level, and conducting cross-sample validation of the adapted item across age groups.

## Data Availability

The data analyzed in this study is subject to the following licenses/restrictions: The dataset used in this study is not publicly available due to ethical and privacy considerations. The data were collected from adolescent middle school students after obtaining formal informed consent, and all variables were measured through self-reported psychological scales. In order to protect participant confidentiality and comply with institutional ethical approval requirements, individual-level raw data cannot be shared in an open-access repository. However, anonymized and de-identified data may be made available by the corresponding author upon reasonable request for academic and non-commercial research purposes, provided that the requesting party agrees to maintain strict data confidentiality and comply with relevant ethical research standards. Requests to access these datasets should be directed to the corresponding author.
